# Efficacy of Intravenous Lidocaine and Magnesium in the Management of Herpes Zoster Neuritis and Postherpetic Neuralgia: A Case Series

**DOI:** 10.7759/cureus.80125

**Published:** 2025-03-06

**Authors:** Shuhei Nagai, Young-Chang P Arai, Keikoi Owari, Makoto Nishihara, Hironori Saisu, Hidemi Niwa, Yuki Terajima, Hiroki Igari, Takahiro Ushida

**Affiliations:** 1 Multidisciplinary Pain Center, Aichi Medical University, Nagakute, JPN

**Keywords:** herpes zoster, intravenous lidocaine, intravenous magnesium, neuropathic pain, postherpetic neuralgia

## Abstract

Objectives: Herpes zoster is a common disease encountered in clinical practice. The neuropathic pain caused by herpes zoster can be severe enough to interfere with daily life in challenging cases. Although it is said that intervening as early as possible after the onset of the disease improves outcomes, there are some cases that are difficult to treat as immediate interventional therapy is not possible. While there are some reports on the efficacy of intravenous magnesium and lidocaine, we investigated the efficacy of intravenous magnesium and lidocaine in patients with herpes zoster neuritis and postherpetic neuralgia.

Patient criteria: A total of 23 patients with herpes zoster neuritis and postherpetic neuralgia were included in the study. We included only cases in which immediate interventional therapy was not possible.

Methods: The treatment protocol was an intravenous infusion of a combination of 1.2 g of magnesium and 100 mg of lidocaine for one hour every week for four weeks (five times in total). Patients were assessed using the Numerical Rating Scale (NRS) for pain before and after the treatment at week zero and before the treatment at weeks one, two, three, and four.

Results: There was a statistically significant difference between the median NRS before treatment at week zero and the median NRS before treatment at week one. Similarly, there was a statistically significant difference between the median NRS before treatment at week zero and the median NRS before treatment at weeks two, three, and four. In addition, the NRS tended to decrease as the number of treatments increased.

Conclusions: Intravenous lidocaine and magnesium may be effective for herpes zoster neuritis and postherpetic neuralgia. It may be a treatment method for when immediate interventional therapy is not possible.

## Introduction

Herpes zoster is a common disease encountered in clinical practice. As the age of the patients increases, the incidence also increases (overall: 3-5/1000 person-years; 60 years: 6-8/1000 person-years; 80 years: 8-12/1000 person-years) [1]; therefore, it is expected that the incidence will increase in the future [2,3]. The neuropathic pain caused by herpes zoster can be severe enough to interfere with daily life in challenging cases [4,5]. Since some research has shown that intervening as early as possible after the onset of the disease improves outcomes [6], we intervened as early as possible. However, there are cases in which immediate intervention is not possible because the patient is taking antiplatelet or anticoagulant medications or has needle phobia [7,8], which can be challenging. Some researchers support the hypothesis that intravenous magnesium produces analgesic effects by acting as an N-methyl-D-aspartate (NMDA) receptor antagonist in the dorsal horn of the spinal cord, while others report that it produces analgesic effects by acting in the peripheral nerves [9,10]. The mechanism of analgesic effects of intravenous lidocaine has been reported in numerous studies. One such theory is that it acts as a sodium channel blocker, achieving analgesic effects by inhibiting nerve conduction [11-14]. In addition, it has been reported that intravenous magnesium added to lidocaine can enhance the analgesic effect more than using intravenous lidocaine alone [15]. Furthermore, we previously reported that intravenous lidocaine and magnesium were effective for the management of intractable trigeminal neuralgia [16]. Thus, we considered that intravenous magnesium and lidocaine might be effective on patients with herpes zoster neuritis and postherpetic neuralgia who cannot be treated immediately by neural blockade due to antiplatelet or anticoagulant medications or needle phobia. In the present case series, we described patients suffering from herpes zoster neuritis and postherpetic neuralgia who were treated with intravenous magnesium and lidocaine.

## Materials and methods

We treated 23 patients (five males, 18 females) with herpes zoster neuritis and postherpetic neuralgia who visited the pain center from September 2021 to November 2023. We included only cases in which immediate interventional therapy was not possible (taking antiplatelet or anticoagulant medications, needle phobia). At our pain center, we primarily treat cases that have not responded to previous treatments and are difficult to manage. As a result, there were no patients who were not taking oral medications at their initial visit. For the patients included in this study, we confirmed that their analgesic medications had not been changed for at least two months prior to the initial visit or since the start of treatment. The treatment protocol was an intravenous infusion of a combination of 1.2 g of magnesium and 100 mg of lidocaine for one hour every week for four weeks (five times in total). Some researchers have used 3-7 mg/kg lidocaine and/or 20-70 mg/kg magnesium for pain management [15,17,18], but a lower and safer dose was chosen in this study. We often use a magnesium ampoule containing 1.2 g magnesium and a lidocaine ampoule containing 100 mg lidocaine, as is the clinical practice in Japan. Furthermore, in a previous study we conducted on intractable trigeminal neuralgia [16], we confirmed the effectiveness of the same method (using an intravenous infusion of a combination of 1.2 g magnesium and 100 mg lidocaine for one hour, once a week) and did not observe any serious side effects. So, we used this protocol in this study as well. Treatment protocols used in the present report were based on institutional policy and clinical guidelines approved by the Institutional Review Board of Aichi Medical University Hospital. The treatment guidelines for patients with intractable neuropathic pain are as follows: patients who are referred to the pain center will be treated while receiving the recommended systemic analgesics, although intravenous magnesium and lidocaine are not indicated for patients with an allergy to lidocaine. We assessed the patients using the Numerical Rating Scale (NRS) for pain, ranging from 0 to 10 (0 = no pain, 10 = worst pain imaginable) before and after the treatment at week zero and before the treatment at weeks one, two, three, and four.

Statistics

We used Windows 11, version 24H2 (Microsoft Corporation, Redmond, WA), and analyzed the data using SPSS version 29.0 (IBM Corp., Armonk, NY) for Windows. We could not confirm equal variances with the Bartlett test. Thus, we performed nonparametric multiple comparisons with correspondence using the Friedman test. Since we found significant differences in that test, we performed the Bonferroni post hoc test. The significance level was set at p < 0.05 for all analyses. In the Bonferroni test, we used the adjusted one.

## Results

Patient demographics

A total of 23 patients (five males and 18 females) were treated. The mean age was 73.3 years. Upon presenting at our clinic, many patients were taking medications such as mirogabalin, pregabalin, and duloxetine (Table 1).

**Table 1:** Characteristics and treatment used for 23 patients with herpes zoster neuritis and postherpetic neuralgia. This table shows patient demographics (gender, age, height, weight, site of pain, pharmacological treatment at the time of the initial visit, and elapsed time from onset of the initial visit). THA: tramadol hydrochloride acetaminophen; MGB: mirogabalin; PGB: pregabalin; AH: amitriptyline hydrochloride; Lt: left; Rt: right.

Gender	Age (years)	Height (cm)	Weight (kg)	Location of pain	Pharmacological treatment (/day)			Elapsed time from onset (in months)
Female 1	68	148	25	C7	MGB 5 mg	Duloxetine 20 mg	Mexiletine 200 mg	4
Female 2	71	153	55	C4	MGB 15 mg	Duloxetine 20 mg	THA 4 tablets	7
Female 3	68	159	80	Rt V1-2	PGB 300 mg		AH 10 mg	2
Female 4	83	152	52	V1-3	MGB 7.5 mg	Duloxetine 20 mg		3
Female 5	68	148	63	V1-3	MGB 30 mg	Duloxetine 20 mg		0.5
Male 1	73	170	60	Th9	MGB 5 mg	Duloxetine 20 mg		3
Male 2	75	164	70	Th10	MGB 5 mg		Mirtazapine 15 mg	1.5
Female 6	83	157	40	Th10	MGB 20 mg	Duloxetine 20 mg		2
Male 3	84	163	63	C2	PGB 150 mg			1
Male 4	71	160	58	Th4	PGB 300 mg	Duloxetine 20 mg		3.5
Female 7	78	155	44	V3	MGB 10 mg	Duloxetine 20 mg		2
Female 8	70	153	60	Th11	MGB 10 mg	Duloxetine 20 mg		1
Female 9	55	153	47	Th5	MGB 10 mg	Duloxetine 20 mg		2.5
Male 5	69	170	76	Rt forearm to hand	MGB 20 mg	Duloxetine 20 mg		2.5
Female 10	71	148	53	Lt back to side	MGB 20 mg	Duloxetine 20 mg		3.5
Female 11	76	155	58	Rt lumbar to abdomen	PGB 75 mg	Duloxetine 20 mg		0.5
Female 12	79	154	53	Lumbar to thigh	PGB 50 mg	Duloxetine 20 mg		1
Female 13	87	160	57	Rt buttocks	PGB 50 mg	Duloxetine 20 mg		6
Female 14	87	154	49	Lt Th4	PGB 100 mg	Duloxetine 40 mg		1.5
Female 15	70	159	59	Rt groin	PGB 100 mg	Duloxetine 20 mg		2.5
Female 16	71	161	60	Rt Th9	PGB 300 mg	Duloxetine 40 mg		2.5
Female 17	74	165	48	Rt Th11	PGB 100 mg	Duloxetine 40 mg		1
Female 18	56	153	47	Lt Th5	MGB 10 mg	Duloxetine 20 mg		2.5

**Figure 1:**
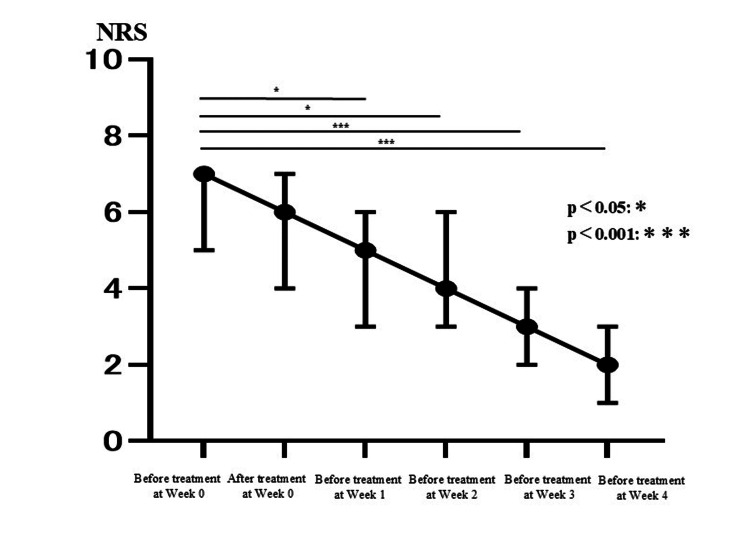
Line chart illustrating the changes in pain scores. With time on the horizontal axis and the Numerical Rating Scale (NRS) on the vertical axis, this figure shows changes in pain scores (median of NRS) over the course of five intravenous treatments. Significant differences are indicated by an asterisk (*).

**Figure 2:**
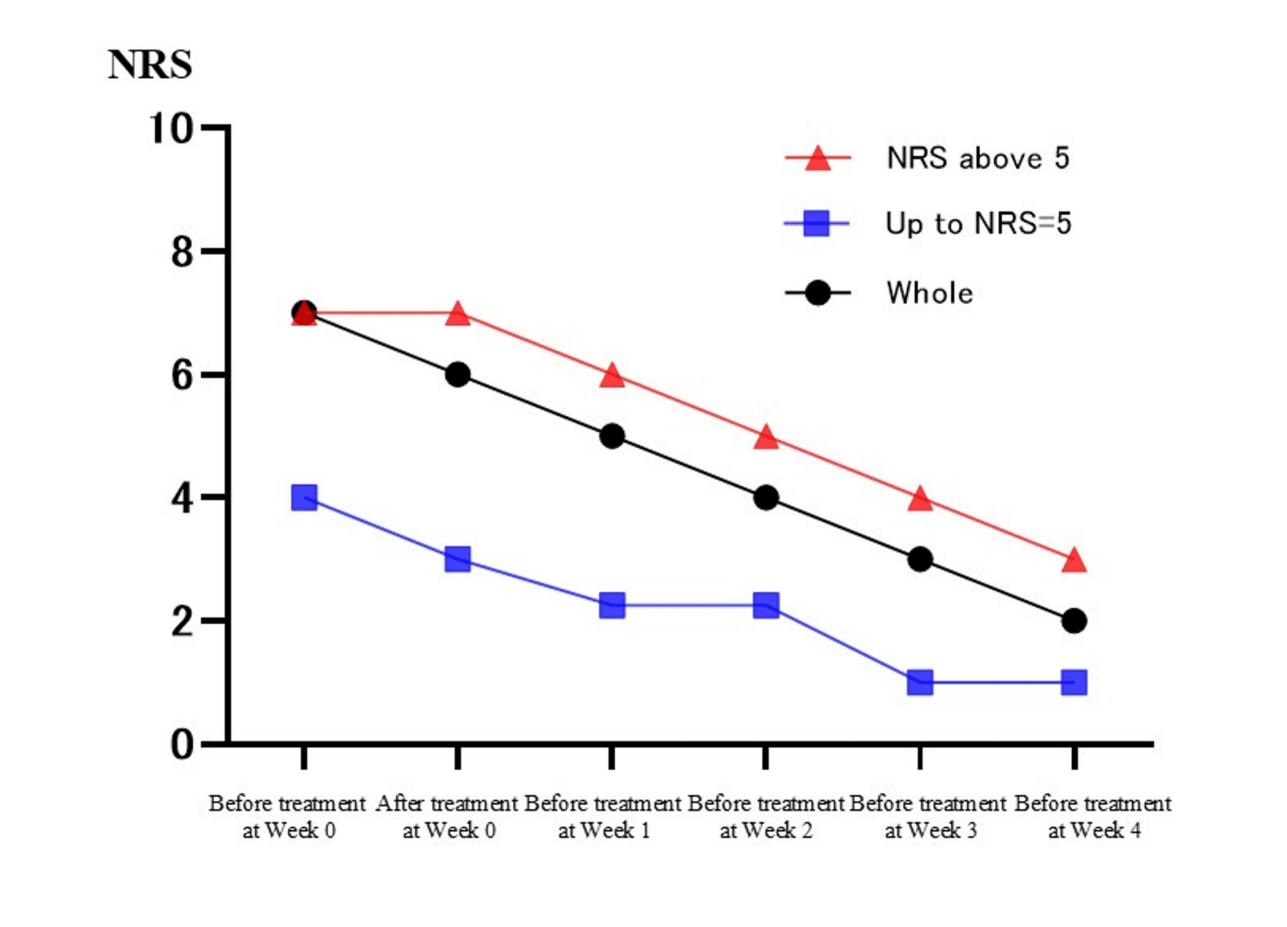
Difference in changes in pain scores between the high NRS group and the low NRS group. With time on the horizontal axis and the Numerical Rating Scale (NRS) on the vertical axis, this figure shows the difference in changes in pain scores between the high NRS group and the low NRS group. The high NRS group was defined as “NRS above 5”, and the low NRS group was defined as “up to NRS = 5”.

**Figure 3:**
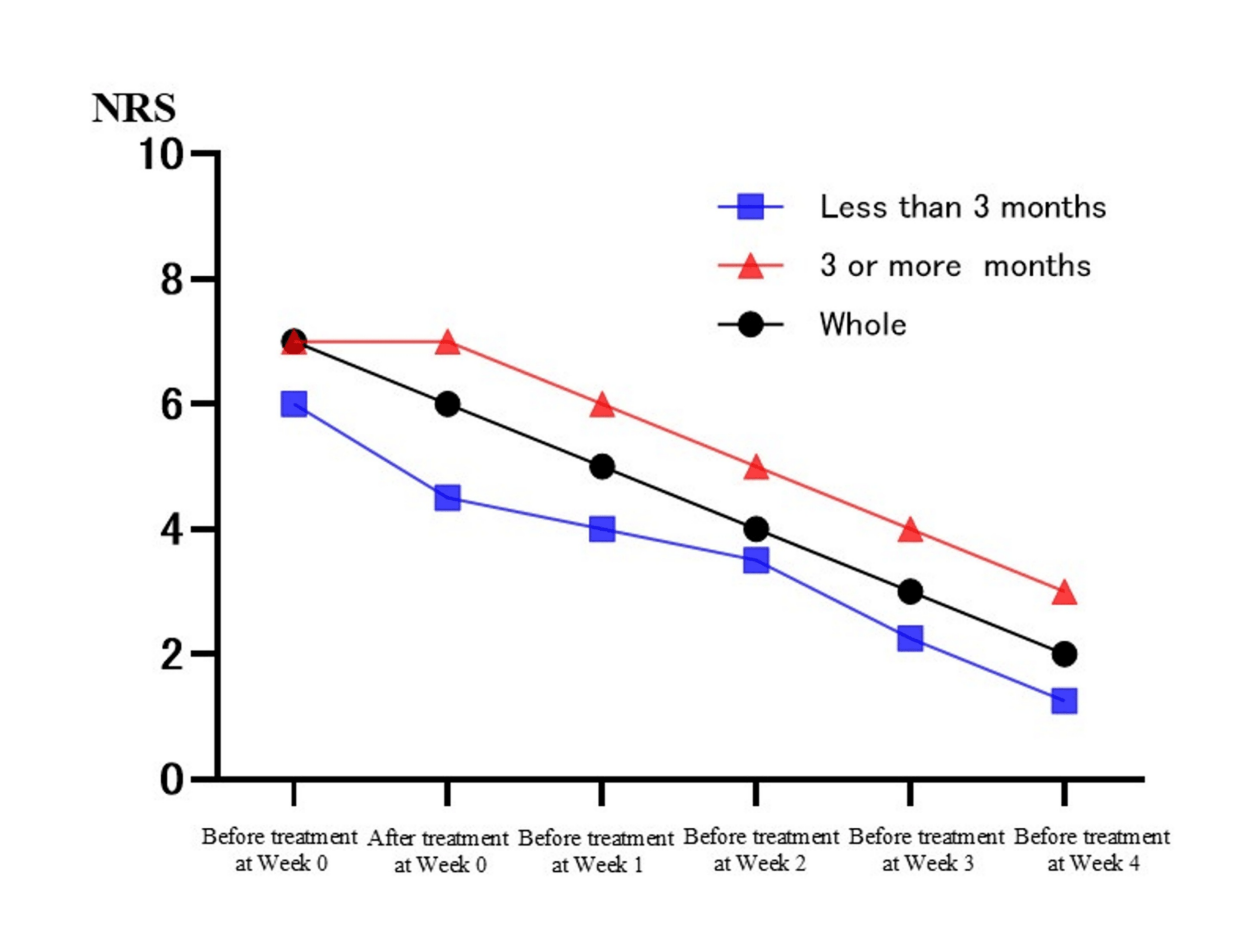
Difference in changes in pain scores between the long disease duration group and the short disease duration group. With time on the horizontal axis and the Numerical Rating Scale (NRS) on the vertical axis, this figure shows the difference in changes in pain scores between the long disease duration group and the short disease duration group. The long disease duration group was defined as "three or more months since onset at their initial visit”, and the short disease duration group was defined as “less than three months since onset at their initial visit”.

Table 2 shows the changes in individual pain scores for the 23 patients. There was a statistically significant difference between the median NRS before treatment at week zero and the median NRS before treatment at week one (median of before treatment at week zero = 7; median of before treatment at week one = 5; Bonferroni test, p < 0.05). Similarly, there was a statistically significant difference between the median NRS before treatment at week zero and the median NRS before treatment at weeks two, three, and four for each (median of before treatment at week zero = 7; median of before treatment at week two = 4; median of before treatment at week three = 3; median of before treatment at week four = 2; Bonferroni test, p < 0.05, p < 0.001). In addition, the NRS tended to decrease as the number of treatments increased. On the other hand, there was no significant difference between the median NRS before and after treatment at week zero (median of before treatment at week zero = 7; median of after treatment at week zero = 6; Bonferroni test, p = 1.00) (Figure 1). Furthermore, we divided the data into two groups (up to NRS = 5 and NRS above 5), as well as into two groups based on the duration of symptoms at their initial visit (three or more months or less than three months since onset). However, no significant differences were found between the groups, and similar trends to the whole results were observed (Figures 2, 3). In this study, there were no side effects reported, and no patients dropped out during the course of the study. In female 2, who had not received interventional therapy after the onset of the disease for seven months, the NRS decreased from 6 to 3 over the course of five intravenous treatments. On the other hand, in female 5 and female 11, who received intravenous treatments the earliest after the onset of the disease (0.5 months), the NRS decreased from 9 to 4 and from 5 to 0, respectively, over the course of five intravenous treatments. In female 4, the NRS did not decrease over the course of five intravenous treatments. Accordingly, she received gamma knife surgery later (Table 2).

**Table 2:** Changes in individual pain scores among 23 patients. This table shows the Numerical Rating Scale (NRS) scores of 23 patients over time.

NRS	Before treatment at week 0	After treatment at week 0	Before treatment at week 1	Before treatment at week 2	Before treatment at week 3	Before treatment at week 4
Female 1	7	4	7	4	4	3
Female 2	6	6	3	3	3	3
Female 3	4	3	3	2	1	0
Female 4	7	7	7	7	7	7
Female 5	9	4	6	6	8	4
Male 1	3	2	3	3	2	2
Male 2	6	6	4	4	3	5
Female 6	8	8	8	8	5	1
Male 3	8	3	8	8	0	0
Male 4	7	7	5	5	4	3
Female 7	7	6	6	6	2	0
Female 8	8.5	8.5	5	5	5	5
Female 9	5	5	2	2	1	1
Male 5	7	7	5	5	2	2
Female 10	8.5	8.5	6	8	5	2
Female 11	5	3	1	3	0	0
Female 12	3	2	1	1.5	0	0
Female 13	9	9	7	8	7	5
Female 14	5	0	2.5	3	2.5	2.5
Female 15	6	6	3.5	3	3	2
Female 16	7	7	4	4	4	3
Female 17	4	4	5	2.5	2.5	1.5
Female 18	4	4	2	2	1	1

## Discussion

Postherpetic neuralgia is defined as pain caused by herpes zoster that persists for at least three to six months after the onset of the rash or continues after the rash has completely healed [19]. It is also reported to be a complication in about 22% of herpes zoster patients [20]. Preventive factors include administration of antiviral drugs within 72 hours of the appearance of the rash, attenuated live herpes zoster vaccine, and early interventional therapy for herpes zoster neuritis [21,22]. Risk factors include age and intensity of acute pain [23]. With an aging population and an increasing number of patients taking anticoagulants and antiplatelet medications, there are some cases in which early therapeutic intervention is difficult. As some reports suggest that deep block injections should be performed at a prothrombin time-international normalized ratio (PT-INR) of less than 1.5 [7], we believe that cases in which nerve blocks are difficult to perform will increase in the future. We believe that intravenous magnesium + lidocaine could be very effective when it is utilized as an early non-interventional therapy. This study showed that the treatment effectively alleviated pain. Furthermore, magnesium and lidocaine are well known to have severe side effects, particularly related to the cardiovascular system, but no side effects were reported in this study. One possible factor for this could be that the doses used were lower than the usual doses. This is also a positive outcome. Thus, we postulate that it will be one of the treatment options for herpes zoster. However, we need to clarify the mechanism and conduct further investigation. Persistent or repetitive painful stimulation of primary nerves in herpes zoster by tissue damage or direct primary nerve damage results in increased reactivity of the spinal dorsal horn nerves. It is said that NMDA receptors play an important role in the mechanism of the wind-up phenomenon, and persistence of this condition leads to central sensitization with increased pain reactivity [24]. In the present case series, we think that lidocaine had analgesic effects by blocking abnormal excitation and conduction of painful nerves as a Na channel blocker. Additionally, we postulate that magnesium had analgesic effects by blocking abnormal excitation of secondary nerves as an NMDA receptor antagonist in the spinal dorsal horn, but analgesic effects in the periphery cannot be ruled out. There is the possibility that a synergistic effect resulted from the combination of two drugs that have different mechanisms of action, and the repeated intravenous treatment might have decreased sensitization gradually, resulting in pain relief.

Limitations

There are several limitations in the present case series. Firstly, we did not see the effects of lidocaine and magnesium as single agents. And the placebo effect cannot be ruled out. We need to study these factors further by conducting a randomized controlled trial. Furthermore, control groups should be included, and confounding factors should be controlled in a better manner.

## Conclusions

Intravenous administration of lidocaine and magnesium appears to be a promising treatment option for patients with herpes zoster neuritis and postherpetic neuralgia, particularly when immediate interventional therapy is not possible due to factors such as anticoagulant use or needle phobia. The treatment demonstrated significant reductions in pain, as evidenced by the reduction in NRS scores after multiple treatments. The combination of lidocaine’s sodium channel blocking properties and magnesium’s NMDA receptor antagonism may offer a synergistic effect, addressing both central and peripheral mechanisms of pain. This treatment approach could serve as an early non-invasive option for pain management in patients who are unable to undergo traditional interventional procedures. However, further research, including randomized controlled trials, is needed to confirm these findings, explore the individual effects of each agent, and better understand the underlying mechanisms of action.
